# MicroRNAs in Nonalcoholic Fatty Liver Disease: Novel Biomarkers and Prognostic Tools during the Transition from Steatosis to Hepatocarcinoma

**DOI:** 10.1155/2014/741465

**Published:** 2014-03-13

**Authors:** Manuele Gori, Mario Arciello, Clara Balsano

**Affiliations:** ^1^Laboratory of Molecular Virology and Oncology, Francesco Balsano Foundation, 00198 Rome, Italy; ^2^Department of Internal Medicine and Medical Specialties, Sapienza University of Rome, 00161 Rome, Italy

## Abstract

Nonalcoholic fatty liver disease (NAFLD) is a metabolic-related disorder ranging from steatosis to steatohepatitis, which may progress to cirrhosis and hepatocellular carcinoma (HCC). The influence of NAFLD on HCC development has drawn attention in recent years. HCC is one of the most common malignant tumors and the third highest cause of cancer-related death. HCC is frequently diagnosed late in the disease course, and patient's prognosis is usually poor. Early diagnosis and identification of the correct stage of liver damage during NAFLD progression can contribute to more effective therapeutic interventions, improving patient outcomes. Therefore, scientists are always searching for new sensitive and reliable markers that could be analysed through minimally invasive tests. MicroRNAs are short noncoding RNAs that act as posttranscriptional regulators of gene expression. Several studies identified specific miRNA expression profiles associated to different histological features of NAFLD. Thus, miRNAs are receiving growing attention as useful noninvasive diagnostic markers to follow the progression of NAFLD and to identify novel therapeutic targets. This review focuses on the current knowledge of the miRNAs involved in NAFLD and related HCC development, highlighting their diagnostic and prognostic value for the screening of NAFLD patients.

## 1. Introduction

**Figure 1:**
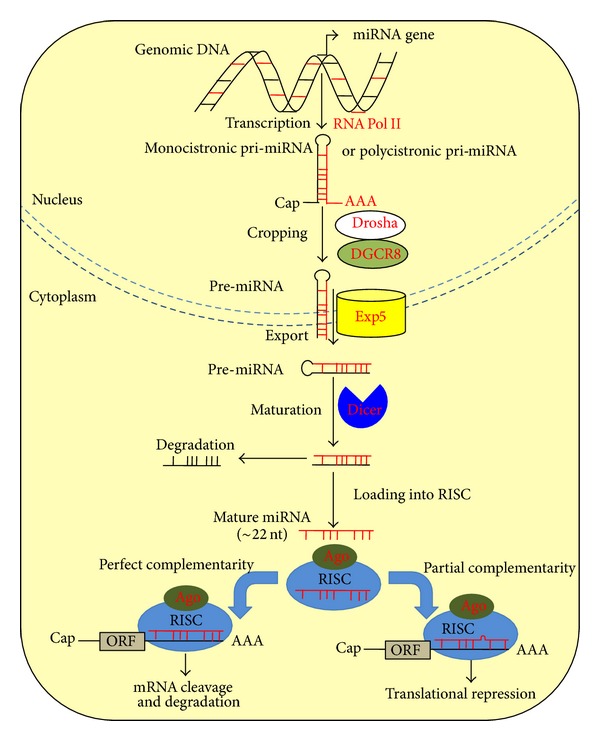
miRNA biogenesis and function. MiRNA genes are transcribed by an RNA polymerase II (RNA Pol II), within the nucleus, as either primary monocystronic or polycistronic transcripts (pri-miRNAs), which are 5′ capped (Cap) and 3′ polyadenylated (AAA). The first processing step (cropping) is mediated by the Drosha-DGCR8 complex (also known as the microprocessor complex) to generate a ~70 nt pre-miRNA folded into a minihairpin structure, which holds a signature motif allowing for recognition by the nuclear export factor, Exportin5 (Exp5). Once exported in the cytoplasm, the RNAse III-like nuclease, Dicer, cuts the loop end of the pre-miRNA in the second processing step (maturation), creating a ~22 nt RNA duplex. The duplex is then separated in two strands of which one is degraded, whereas the other strand is selected as the mature miRNA that is loaded into the RNA-induced silencing complex (RISC), in the final step of miRNA biogenesis. This final effector complex is composed of the core protein, Argonaute (Ago), which is required for the pairing between the miRNA and target mRNAs. Depending on the degree of complementarity with the 3′-UTR of target mRNA, the mRNA is either subject to translational repression (if partial complementarity occurs) or cleavage and degradation (if perfect complementarity occurs), with the final result of mRNA silencing [26, 127].

Nonalcoholic fatty liver disease (NAFLD) is the most common chronic liver disease in developed countries [1]. It ranges from simple steatosis, the intrahepatic fat accumulation exceeding 5–10% by weight, to the more aggressive nonalcoholic steatohepatitis (NASH), which may progress to cirrhosis and, in a small percentage of patients, to hepatocellular carcinoma (HCC) [1, 2]. The presence of hepatic steatosis may represent a risk factor for HCC development. Even if the prevalence of HCC in NAFLD patients is 0%–0.5%, it is expected that its incidence and prevalence will also increase [1, 3]. NAFLD is considered a multifactorial disease and the hepatic manifestation of the metabolic syndrome (MeS) [4, 5]. In developed countries, the incidence of NAFLD is between 10% and 30% [6], reaching 60% to over 90% among obese [7]. Obesity, diabetes mellitus, elevated glucose, hyperlipidemia, MeS, and insulin resistance (IR) have been established as risk factors for NAFLD [1]. Although a long-term evaluation of the NAFLD outcome is lacking, patients with isolated steatosis on presentation generally have a benign prognosis [8]. NASH is observed in a subset of NAFLD patients and is characterized by variable degrees of hepatocyte damage, inflammation, and fibrosis [2]. In a small percentage of patients NASH can progress to end-stage liver disease, such as cirrhosis and HCC (0%–2.8%) over a 19.5-year period [1], but regression is also possible in precirrhotic stages [9]. Since some of the histologic hallmarks of NASH, such as fatty deposition and necroinflammation, may disappear when the disease progresses to cirrhosis [1], making a definitive diagnosis difficult, the identification of new prognostic markers of HCC is of primary importance. Even though cirrhosis is the most important single risk factor for HCC and is present in about 80% of patients with HCC, regardless of the underlying liver disease [10], rare but increasing cases of HCC arising in NAFLD patients without cirrhosis raise the possibility that carcinogenesis secondary to NAFLD can occur in the absence of advanced liver disease [11]. Thus, in a prospective setting it is essential for the scientific community to quantify the risk and develop surveillance recommendations for detection of HCC with MeS, both with and without cirrhosis [11]. At present, it is not yet possible to predict the NAFLD outcome through the routinely used blood and tissue biomarkers due to their limited prognostic usefulness, tissue specificity, sensitivity, diagnostic accuracy, and sometimes misleading criteria of evaluation in patients with liver inflammation/fibrosis during the different stages of hepatic disease. Thus, liver biopsy still represents the gold standard for the diagnosis and staging of NAFLD and hepatocarcinoma. In fact, only microscopic evaluation of an adequate biopsy is able to provide additional information on the presence and extent of ongoing necroinflammatory injury, localization of fibrosis, and extent of parenchymal remodeling. Liver biopsy evaluation also shows that a still unknown percentage of individuals with initial diagnosis and clinical manifestations of NAFLD may not have steatohepatitis or even steatosis [12]. Notwithstanding such advantages, this method is invasive, potentially dangerous, expensive, and limited in accessibility and reproducibility as well [13, 14]. Furthermore, NAFLD encompasses a wide spectrum of liver damages; thus, there is a lack of absolute positive or negative markers to study the disease progression [15] and the onset of HCC [16]. Moreover, prognosis of HCC, which is considered a multifactorial malignancy, is not solely dependent on tumour burden but is also adversely influenced by impaired liver function secondary to the underlying pathogenic condition [1, 10, 16]. Ideal biomarkers to follow the evolution of NAFLD to HCC should possess high specificity and sensitivity. They should be easily accessed and measurable, minimally invasive, inexpensive to quantify, accurate, and acceptable to patients and physicians [17]. In addition, they have to be validated in different populations and possibly by independent external investigators. The lack of a precise date of initiation of the disease process, the uncertainty of which endpoint to measure for each stage of the disease, a proper reference standard, and optimal cutoff values among different laboratories are significant matters of debate. For instance, the variety of methods of histologic analysis by immunohistochemical staining in NAFLD/NASH specimens may have limitations due to discretionary accruals and bias among different centers. The argument of which stage or stages of NAFLD require diagnosis, attempting to distinguish NASH from simple steatosis, or fibrosis from NASH, and so forth may have a great impact on the choice of the optimal noninvasive markers. Finally, a major problem for a proper diagnosis/prognosis is choosing the correct study population (using the same methods of patient recruitment and exclusion criteria), also because of the requirement of liver biopsy as the reference test with the risk of a certain degree of selection bias. In spite of the number of the currently used biomarkers to assess HCC occurrence in NAFLD patients, such as alpha-fetoprotein (AFP), alone or in combination with additional tissue and serum markers, Osteopontin, Glypican-3 (GPC-3), Hyaluronic acid, Laminin, VEGF [18–22], and so forth, their usefulness still remains limited due to lack of sensitivity, specificity, and predictive values for a wide population screening. For this reason, a growing number of studies are focused on the identification and validation of novel and trustworthy biomarkers for creating more exhaustive panels. MicroRNAs (miRNAs) are endogenous 19–24 nucleotides noncoding single-stranded RNAs that control, at posttranscriptional level, many complementary target mRNAs implicated in several pathophysiological processes, such as cell proliferation, differentiation, metabolism, apoptosis, and cancer [23]. First discovered in* Caenorhabditis elegans*, in 1993, they were subsequently identified also in plants and animals, up to humans [23]. To date, the human genome is predicted to encode ~1000 miRNAs, and it is estimated that they can regulate approximately one third of all human transcripts [24, 25]. MiRNAs have been found in different genomic locations: intergenic miRNA genes, intronic miRNAs, within both protein coding genes and long noncoding RNAs, and the far more rare exonic miRNAs. Notably, some miRNAs are expressed in the same direction as neighboring protein-encoding genes and in parallel with their host transcripts, whereas some are antisense to neighboring genes [26, 27]. First transcribed as pri-miRNAs, these molecules are further processed in two steps, within the nucleus and then in the cytoplasm, to form mature miRNAs. The mature forms, finally exert either the silencing of target mRNA species or the repression of protein synthesis [24] (Figure 1). MiRNAs have been suggested as the ideal class of blood-based biomarkers for cancer detection [28]. They have shown specific expression in different body fluids and to be dysregulated in liver diseases, from simple steatosis to HCC [23, 29–31]. Thus, their analysis, in combination with other clinical parameters and standard liver examinations, may be extremely useful to predict the possible progression of NAFLD up to HCC and for monitoring response to treatments.

## 2. Tissue and Circulating miRNAs as Promising Biomarkers for Predicting NAFLD Progression and HCC Development

Nowadays, miRNAs are receiving growing attention also because they are frequently dysregulated in a variety of pathological conditions, including liver diseases [23], and have shown to be novel tissue-based markers for the diagnosis and monitoring of several tumors, including HCC [29, 32, 33]. In fact, some of them, and their processing components such as the enzymes Dicer1, Drosha, microprocessor complex subunit DGCR8, and RISC-loading complex subunit TARBP2, have shown to be downregulated in multiple cancers. Ablation of miRNA processing enzymes in cancer cells promotes tumor invasiveness and more aggressive phenotypes. These characteristics reveal their essentiality in controlling tumor- and metastasis-initiating events [34]. Based on their wide range of potential roles in carcinogenesis, miRNAs are categorized as either oncogenes (oncomiRs) or tumor suppressors. MiRNAs have been recently shown to be stably expressed in serum, plasma, urine, saliva, and other body fluids, and their patterns of expression are linked to various types of human tissue injury and cancer. Therefore, serum or plasma miRNAs are now emerging as a class of useful and specific noninvasive biomarkers for a broad range of liver diseases and for the early detection of HCC [29–31]. Lately, microRNAs were suggested to play an important role also in NAFLD [31, 35–37]. They are key regulators of cholesterol and fatty acid (FA) metabolism [38] and are able to modulate target molecules and pathways known to be involved in NAFLD pathogenesis and progression, such as interleukin-6 (IL-6) and tumor necrosis factor-*α* (TNF-*α*). Moreover, NAFLD progression can be facilitated by soluble factors and signaling molecules, such as cytokines and adipokines, secreted by visceral adipose tissue (VAT). Among the various miRNAs linked to NAFLD/NASH, some that are produced by VAT showed different expression patterns depending on the subtypes of NAFLD. For instance, the analysis of Estep et al. revealed a significant association between the miRNAs differentially expressed in NASH and some pathways involved in the occurrence of HCC and cardiac diseases [39]. Interestingly, another group observed an upregulation of the miRNA processing machinery components (i.e., Dicer1, Drosha, and DGCR8), along with the expression of specific pri-miRNAs (such as pri-miR-7-1, pri-miR-16-2, and pri-miR-26a-1) in VAT samples from NASH, compared to non-NASH/NAFLD patients; hence, their findings suggest that VAT-derived miRNAs may contribute to the pathogenesis of NASH in obese subjects [40]. However, despite the association between definite miRNA signatures and pathogenesis of NAFLD, the expression levels of specific hepatic miRNAs during NAFLD are still controversial [35–37], and further investigations are required to shed light on their function in the context of NAFLD.

Herein, we will summarize the expression pattern of some of the most common miRNAs involved in hepatic lipid metabolism and disease, since their number has been continuously increasing, and will discuss their great potential as diagnostic and prognostic biomarkers of NAFLD and NAFLD-related cirrhosis and HCC (Table 1).

### 2.1. MiR-122

MiR-122 is the most expressed miRNA in adult human liver (approx. 70% of total miRNAs) and the first described for its role in regulating total serum cholesterol and hepatic lipid metabolism [41]. Moreover, miR-122 appears to maintain the hepatic cell phenotype, and its inhibition decreases total serum cholesterol and FA synthesis [41, 42]. Related to miR-122, miR-370 has also been identified as another key regulator of lipid metabolism, which can indirectly upregulate lipid synthesis by activating miR-122 [43]. For all these reasons, miR-122 has been suggested as a possible therapeutic target in the treatment of hypercholesterolemia in humans [44], such that it is in phase I clinical trials for treating dyslipidemia [42]. Cheung and coworkers identified 46 miRNAs differentially expressed in subjects with MeS and NASH versus those with normal liver histology [35]. They found 23 microRNAs differentially modulated and, in particular, that miR-122 expression was significantly decreased in NASH patients. They correlated its downregulation to the alteration of hepatic lipid metabolism genes, implicated in NASH development, such as fatty acid synthase (FAS), 3-hydroxy-3-methylglutaryl-CoA reductase (HMGCR), sterol regulatory element-binding protein-1c (SREBP-1c), and sterol regulatory element-binding protein 2 (SREBP-2). On the contrary, a recent work showed that in a cohort of 34 NAFLD patients miR-122 levels were positively associated with disease severity, from simple steatosis to steatohepatitis. In particular, its serum levels correlated with serum lipids, fibrosis stage, and inflammation activity [31], and similar results were found in mouse models of diet-induced NAFLD [45]. The link between miR-122 and the grade of liver steatosis was further strengthened by a recent study undertaken in a cohort of NAFLD patients, in which the authors found a positive correlation between miR-122 serum levels and the severity of steatosis, concluding that miR-122 may be a useful biomarker for NAFLD progression. Interestingly, this correlation appears to be independent of patient's gender [46]. Thus, miR-122 may potentially serve as circulating marker for assessing the stage of liver disease. Unfortunately, it does not seem to be NAFLD specific, because similar results were also reported in patients affected by chronic hepatitis C (CHC) [31]. The repression of miR-122 in HCC, which is a tumor suppressor miRNA, has been reported by many investigators [47, 48]. Additionally, miR-122 has been identified as an inhibitor of AFP expression and of aggressive features in HCC [49]. Based on this, the routinely evaluation of miR122 may lead to the definition of a cutoff point that could be useful to identify the stage of disease progression, thus becoming a reliable biomarker for various liver diseases. Interestingly, an inverse correlation has been shown between circulating and tissue/cell levels of miR-122. In fact, its level was decreased in the liver tissue but increased in the plasma or serum of patients with cirrhosis, HCC, and hepatitis B virus (HBV) infection [29, 50, 51]. A similar phenomenon was found between cultured HCC cells and culture medium. This inverse correlation supports the possibility of using circulating miRNAs as fingerprints for a diagnostic and prognostic evaluation of the disease [29].

### 2.2. MiR-34a

MiR-34a is known to be increased in fatty livers of diet-induced obese mice [52] and in serum of NAFLD patients [46], in which it correlates with the severity of the disease. Indeed, miR-34a has been shown to be implicated in the dysregulation of cholesterol metabolism by targeting the hepatic NAD-dependent deacetylase Sirtuin 1 (SIRT1), which is critically involved in the modulation of liver cell apoptosis, metabolic disease, and cancer [52, 53]. A recent study revealed an interesting link between the progression of rat and human NAFLD and NASH and miR-34a/SIRT1/p53 signaling, which is specifically modulated by the exacerbation of inflammation, triggering in turn hepatocyte apoptosis [54]. Thus, miR-34a expression in the human liver significantly increases with NAFLD severity, confirming its usefulness as a novel and noninvasive biomarker. In 2008 a validation analysis, by microarray assay of differentially expressed miRNAs in NASH subjects versus controls, found miR-34a and miR-146b to be significantly overexpressed in liver of NASH subjects, correlating with steatohepatitis development [35]. Recently, plasma levels of miR-34a, together with miR-122, miR-181a, miR-192, and miR-200b, were shown to be significantly associated with the severity of NAFLD-specific liver pathomorphological features in mice, and miR-34a had the strongest correlation [45]. Accordingly, they all can be used for noninvasive monitoring the susceptibility and the extent of NAFLD. A positive correlation between serum levels of miR-34a and disease severity was also demonstrated in both CHC and NAFLD patients, as well as in NASH patients in which miR-34a was overexpressed [31]. Differences in the expression of hepatic miR-34a and other miRNAs were shown to be associated with susceptibility and severity of dietary NASH in mice, arguing that these miRNAs could be valuable steatohepatitis biomarkers [55]. Thus, miR-34a may represent a useful, noninvasive biomarker of diagnosis and histological disease severity in patients, which correlates with the levels of liver enzymes, fibrosis stage, and inflammation activity. The relevance of miR-34a in cancer was highlighted by a work of Budhu and colleagues, on a series of miRNAs as a HCC metastasis signature to predict survival and recurrence of hepatocarcinoma [56]. They showed that miR-34 was highly downregulated in metastasis cases, representing an independent and significant predictor of patient prognosis when compared to other available clinical parameters. These data are in agreement with experimental investigations revealing that miR-34 family members (miR-34a,b,c) are also direct transcriptional targets of the tumor suppressor p53 [57, 58]. This mechanism is important not only for tumorigenesis, because loss of miR-34 function can impair p53-mediated cell cycle arrest and apoptosis [58], but also for NAFLD. In fact, recent papers reveal that p53, as pivotal metabolic modulator, may play key roles in lipid metabolism [52, 59] and in the pathogenesis and progression of NAFLD [60, 61]. In this regard, it has been recently proposed a model in which pharmacological inhibition of p53-driven miR-34a upregulation attenuates steatosis and liver injury in NAFLD mice [60]. Finally, the p53/miR-34 axis is at the core of a regulatory network targeting the oncogenic Wnt signaling pathway, responsible for the snail family zinc finger 1 (Snail1) protein-dependent epithelial-mesenchymal transition (EMT) program in a miR-34/UTR-specific manner [62]; EMT, in fibrotic and cirrhotic livers, has important implications for the progression to HCC [63, 64]. In this scenario, a detailed analysis in human cancer cell lines demonstrated that the reciprocal repression of miR-34a and Snail1 finely regulates the EMT/mesenchymal-epithelial transition (MET) balance. These studies have provided further insight into the complex mechanisms of the double-negative feedback loop, involving p53/miR-34 and Snail, which controls EMT as well as Cancer Stem Cell (CSC) plasticity, migration, and invasion in hepatic fibrosis and tumors [62].

### 2.3. MiR-16

MiR-16 is a 21-nucleotide miRNA that was found overexpressed in rat and human NAFLD/NASH, when compared to healthy subjects, correlating with the progression of liver inflammation [35]. Circulating levels of miR-16, together with miR-34a and miR-122, may potentially be useful biomarkers for the assessment of disease stage in NAFLD subjects, as suggested by the study of Cermelli et al. in which these miRNAs were significantly higher in patients than in control individuals and linked with the severity of the diseases [31]. During the progression of NASH towards HCC, a marked upregulation of miR-16 has also been observed [40, 65]. However, other studies carried out by Guo and colleagues found miR-16 and miR-15b downregulated during the activation of hepatic stellate cells (HSCs), as also confirmed by further* in vitro *experiments in which their upregulation significantly inhibited HSC proliferation, by increasing their apoptosis levels, and reduced hepatic fibrosis [66]. The scientific literature emphasises the importance of miR-16 in NAFLD pathogenesis and supports its possible candidature as valuable prognostic marker for the disease [31, 35, 40]. Although the findings obtained in CHC patients reveal that miR-16 might not be a specific marker for NAFLD [31], its combination with additional and more specific miRNAs could help achieve more useful diagnostic/prognostic results.

### 2.4. MiR-33a/b

MiR-33a/b are the most known miRNAs to promote cholesterol and FA biosynthesis in both mouse and human [42]. These findings were also confirmed in nonhuman primates, where their expression was associated with decreased plasma levels of very-low-density lipoprotein (VLDL)-associated triglycerides and increased high-density lipoprotein (HDL)-associated triglycerides [67]. In addition, miR-33a/b control the expression of genes involved in hepatic FA oxidation (FAO) and insulin signaling and reduce the expression of genes involved in FA synthesis. Indeed, inhibition of miR-33a/b in hepatic cell lines has been shown to increase both FAO and insulin pathways that along with lipogenesis may represent risk factors for the development of MeS [68]. Hence, miR-33a/b are considered promising therapeutic targets against MeS and atherosclerosis. Besides, miR-33 inhibition was shown to improve liver regeneration in mice, whereas its overexpression in human hepatoma cell lines induced cell cycle arrest [69], leading researchers to speculate an important role for miR-33 also in human liver regeneration. Thus, miR-33a/b, together with other miRNAs, may play key roles in the onset and progression of hepatic metabolic disorders, including NAFLD, representing additional useful biomarkers to follow the progression of the disease up to HCC in which they are upregulated [70].

### 2.5. MiR-200

The miR-200 family contains miR-200a, miR-200b, miR-200c, miR-141, and miR-429. Among them, miR-200a, miR-200b, and miR-429 were found to be upregulated in rat models of hypercaloric diet-induced NAFLD, compared to controls. The dysregulated expression was associated with increased body weight and altered histological and metabolic parameters characteristic of NAFLD. Interestingly, these miRNAs were found to target various molecules involved, among others, in apoptosis, lipid, and carbohydrate metabolism pathways, whose proteins showed a deranged expression in the hypercaloric diet-fed animals [71]. The association between miR-200b expression and pathophysiological features of NASH was demonstrated in livers of a mouse model similar to human NASH. Mice fed with lipogenic methyl-deficient diet displayed a specific differential expression pattern of miRNAs and their target genes, compared to controls [55]. The development of NASH was paralleled by overexpression of miR-200b along with miR-34a, miR-155, and downregulation of miR-29c, suggesting that severity of and susceptibility to diet-induced NASH may be determined by alterations in these specific miRNAs expression [55]. Similar findings were also obtained in diverse strains of mice fed a choline- and folate-deficient diet [45]; in this work, circulating levels of a group of miRNAs, including miR-200b, significantly correlated with severity of NAFLD-specific liver damage, thus representing highly sensitive plasma biomarkers that allow noninvasive monitoring of the progression and extent of NAFLD-related liver injury. In the study of Murakami et al. [72], in both mouse models and human clinical specimens of liver fibrosis, the expression of 11 miRNAs was related to the progression of the disease; notably, the upregulation of four miRNAs, miR-199a, -199a*, -200a, and -200b, positively and significantly correlated with the grade of fibrosis. Furthermore, their overexpression in HSCs significantly increased the level of fibrosis-related genes. Altogether, these data underline the potential of the miR-200 family as therapeutic and diagnostic tool for hepatic fibrosis. Much is known about the role of the miR-200 family in HCC metastatic pathways. In fact, it was demonstrated that in different HCC cell lines, miR-200a and miR-200b control their migratory ability by targeting the expression of the cell-cell adhesion molecule E-cadherin [73]. Recently, Zhang and colleagues have deepened this topic [74], concluding that the miR-200 family could be a possible therapeutic target against hepatocarcinoma metastasis. In their work they showed, both* in vitro* and* in vivo*, that epigenetic activation of the miR-200 pathway by the long noncoding RNA H19 reverses the EMT response, thus suppressing the rate of tumor metastasis also in aggressive HCC.

### 2.6. MiR-99a/b

The miR-99a/b is a family of tumor suppressor miRNAs. MiR-99a was found to be the sixth most abundant microRNA in the miRNome of normal human liver but was markedly downregulated in HCC, playing an important role in the inhibition of tumor growth by inducing cell cycle arrest. Accordingly, miR-99a has been proposed as a prospective predictor of HCC prognosis [75]. MiR-99a/b downregulation in adipose tissue of obese and NAFLD patients has been widely demonstrated; miR-99a showed its negative correlation with serum levels of free fatty acids (FFAs) and IL-6 [39, 76]. In addition, the expression of miR-99b together with miR-197, both of which are secreted by VAT of NAFLD patients, has been observed to be significantly associated with pericellular fibrosis in NASH patients. Thus, miRNAs expression from VAT may represent a possible new means for distinguishing between simple steatosis and NASH [39].

### 2.7. MiR-21

As far as the involvement in liver steatosis, miR-21 was found to be downregulated in the livers of ob/ob mice together with miR-29c and miR-451, suggesting a possible role for these miRNAs in NAFLD, which needs to be addressed in more detail [36]. An interesting report strengthens miR-21 association with steatosis showing that lycopene (an unsaturated carotenoid antioxidant) inhibits hepatic steatosis* via* miR-21-induced downregulation of its downstream target, the fatty acid-binding protein 7 (FABP7), in mice fed a high-fat diet [77]. However, in the work of Cermelli et al., serum levels of miR-21 were found to be unchanged between NAFLD patients with fibrosis and healthy controls unlike miR-34a and miR-122, which instead may have utility in the identification of those NAFLD patients who developed significant liver fibrosis. These latter, indeed, correlated with the grade of steatosis, fibrosis stage, and inflammation [31]. A recent investigation of Yamada and coworkers showed that serum levels of miR-21 were significantly higher in men with NAFLD than controls, but no statistically significant difference was observed between levels of miR-21 in women participants [46]. The evaluation of its association with other known oncomirs related to liver steatosis revealed that unlike the serum levels of miR-122, those of miR-21, miR-34a, and miR-451 increased with disease severity, although the correlations were not statistically significant in participants of both sexes [46]. MiR-21 is one of the first miRNAs identified and described as an oncomir. It is now considered the prototype oncomir, with most of its targets known to be tumor suppressor genes, such as phosphatase and tensin homolog (PTEN), acidic (leucine-rich) nuclear phosphoprotein 32 family, member A (ANP32A), SWI/SNF related, matrix associated, actin dependent regulator of chromatin, subfamily A, and member 4 (SMARCA4) [33, 78]. In particular, among them, PTEN has been reported to play a key role in the development and progression of NAFLD, leading to HCC [33, 79]. Indeed, it has been shown that hepatocyte-specific downregulation of PTEN in steatotic rat and human livers is mediated by high levels of unsaturated fatty acids* via* activation of a nuclear factor kappa B (NF-*κ*Bp65)/mammalian target of rapamycin (mTOR)-dependent mechanism. The same pathway, triggered by dietary FFAs, is also responsible for promoting hepatoma proliferation and carcinogenesis [80]. Further studies carried out by Vinciguerra and coworkers unraveled the molecular mechanism which inhibits PTEN expression in the liver of both high-fat diet-fed rats and obese patients with steatosis [81]. In this work they found an upregulation of miR-21 in hepatocytes, mediated by unsaturated fatty acids, through the activation of the mTOR/NF-*κ*B complex. MicroRNA-21 in turn, by specifically binding to the 3′-UTR of the PTEN mRNA, induced its degradation. This evidence thus further supports the role of miR-21 as useful biomarker in NAFLD. MiR-21 is also upregulated in liver fibrosis [82] and commonly deregulated in HCC along with miR-122, miR-34a, and miR-16 [83, 84]. In fact, miR-21 was identified to be consistently upregulated in sera and plasma from patients with different cancers, including HCC [83, 84], making it an interesting candidate as serum/plasma marker for malignancies. A recent analysis in patients with primary HCC revealed that high levels of miR-21, together with miR-31, miR-122, and miR-221, were correlated with cirrhosis, but there was no correlation with HBV and HCV infection. Particularly, the expression levels of miR-21 and miR-221, evaluated by multivariate Cox regression analysis, were correlated with tumor stage in HCC patients, hypothesizing that miR-21 and miR-221 levels might affect the prognosis of HCC and might be independent predicting factors in the overall survival of HCC patients [85].

### 2.8. MiR-221

Interestingly, miR-221 is one of the miRNAs upregulated in the livers of ob/ob mice, suggesting an important role in NAFLD [36]. The expression pattern of miR-221, in mouse and human livers was also shown to be consistent with fibrosis grade being upregulated with fibrosis progression and thus involved in the regulation of liver fibrosis contrary to miR-29 members, which were significantly downregulated in fibrosis and advanced cirrhotic patients [86]. Accordingly,* in vitro* and* in vivo *studies proposed miR-221/222 as new markers for the activation of HSCs and the progression of liver fibrosis [87]. This miR-221/222 family of hepatic miRNAs was found to be dysregulated at early stages of NASH-induced HCC in mice and involved in hepatocarcinogenesis [88]. These studies highlight the relevance of miR-221 in NAFLD pathogenesis and its potential prognostic value for disease progression. MiR-221 is considered an oncomir; it is one of the most upregulated in HCC, in both a transgenic mouse model [89] and in humans, where about 70–80% of the examined HCC showed its upregulation [90]. Furthermore, its serum and tissue levels were shown to correlate with cirrhosis, higher tumor stage and size, metastasis, and lower survival rate in HCC patients versus healthy controls [85, 91], confirming its diagnostic and prognostic significance, as well as its possible role as a target in liver cancer therapy.

### 2.9. MiR-155

The expression of MiR-155 has been associated to NAFLD progression; it is indeed one of those miRNAs (including miR-200b and miR-34a) upregulated in mouse models of diet-induced liver injury similar to human NASH, which is considered to be relevant for and correlated to the development and severity of the disease [55]. In a microarray analysis conducted on mice fed a choline-deficient L-aminoacid-defined diet, miR-155 was found to be upregulated at early stages of NASH-induced hepatocarcinogenesis, as well as in primary human HCCs compared to matching liver tissues [88]. In addition, this miRNA is thought to play an important role in regulating both inflammation and tumorigenesis; in fact, it was shown to be upregulated in mouse and primary human HCCs and to target the transcription factor CCAAT/enhancer binding protein beta (c/EBP*β*), which is downregulated at early stages of hepatocarcinogenesis in mice and humans [88]. A recent research has demonstrated a gradual ascension of miR-155 expression in cirrhotic and HCC tissues, compared with low expression levels detected in normal liver tissues [92]. Thus, increased expression of miR-155 enhances liver cell tumorigenesis, and this finding suggests that this oncogenic miRNA may be a potential target for HCC treatment.

### 2.10. MiR-181a/b

In a recent work on mouse models of NAFLD, plasma levels of miR-181a were found to be associated with susceptibility to NAFLD and the extent of NAFLD-associated liver injury [45]. Lately, miR-181b, but not miR-181a, has been proposed as a new potential diagnostic serum marker of liver cirrhosis in humans [93]. In fact, in this study, miR-181b was shown to be induced by transforming growth factor-*β*1 (TGF-*β*1) and to promote HSCs growth by directly targeting the cyclin-dependent kinase inhibitor 1B (p27). Its serum levels resulted to be elevated in cirrhotic patients compared to normal subjects. MiR-181 was found to be upregulated also in CD133^+^, epithelial cell adhesion molecule (EpCAM)^+^, and AFP^+^ Tumor-Initiating Stem Cells (TISCs) by miRNA expression profiling in HCC. This upregulation was driven by the upmodulation of the wingless (Wnt)/*β*-catenin pathway, which is known to play a critical role in CSCs origin and malignancy in benign adenomas and also modulated through the activation of the *β*-catenin target oncogene c-Myc [94–96]. Moreover, a recent analysis carried out in hepatocellular CSCs,* via* global microarray-based microRNA expression profiling and RT-PCR validation, showed that conserved miR-181 and let-7 family members, whose expression is regulated by IL-6 and the basic helix-loop-helix transcription factor Twist, were upregulated in these cancer cells. In the same study, it was demonstrated that these families of miRNAs play an important role in tumour spreading and following their inhibition in the responsiveness to chemotherapy [97].

### 2.11. MiR-10b

An* in vitro* study of the expression profiling of miRNAs in steatotic human hepatocytes led to the identification of miR-10b as a new important regulator of steatosis levels, uncovering a novel mechanism for NAFLD pathogenesis [98]. In fact, it was identified the effect of miR-10b on posttranscriptional regulation of the nuclear receptor peroxisome proliferator-activated receptor-*α* (PPAR-*α*), which is involved in storage and catabolism of FAs and liver inflammation participating in steatosis. In steatotic hepatocytes miR-10b was upregulated, and its overexpression increased intracellular triglyceride levels and lipid content by targeting PPAR-*α* [98]. These results suggested that miR-10b is another potential therapeutic target for the treatment of NAFLD. Its deregulated expression in some types of cancer [99, 100] prompted scientists to consider this miRNA as a critical target in neoplastic transformation as well, which holds therapeutic promise. Actually, it has been highlighted its involvement in the establishment (miR-10b*), migration, and spreading (miR-10b) of human breast cancer, by controlling cell cycle progression and proliferation [101]. In particular miR-10b, highly expressed in metastatic HCC tissues and cell lines, constitutes an independent predictor of poor prognosis in patients, promoting migration and metastasis in human hepatocarcinoma cells [99].

### 2.12. Let-7

In the context of NAFLD, there is a peculiar regulation of the expression of different members of the let-7 family. In fact, contrary to let-7b, which was found to be upregulated in steatohepatitis compared to steatosis samples, let-7d was instead downregulated in the former; these data imply a diverse modulation, among the various let-7 family members, during the progression of NAFLD, and also a role for them as predictive biomarkers [102]. The let-7 family is well recognized to play pivotal roles during liver fibrosis and tumorigenesis, by protecting human hepatocytes from oxidative stress and functioning as potential suppressor of cell growth [103, 104]. For example, it has been recently reported that indirect upregulation of heme oxygenase 1 (HMOX1) gene expression, which is a key cytoprotective enzyme, by let-7 miRNAs (let-7b,c and miR-98) attenuates oxidant injury in human hepatocytes. Hence, overexpression of specific let-7 family members may represent a novel approach to protecting hepatocytes from oxidant injury, which represents an important step in the progression and acceleration of several liver diseases, such as fibrosis and HCC [104]. On the contrary, the expression pattern of let-7e, another member of this family, was related to the progression of liver fibrosis in mouse models [72]. As far as the involvement of the let-7 family in liver cancer, the expression levels of let-7c, analysed in 32 HCC patients, were significantly lower in HCC tissues than in the corresponding normal adjacent tumour tissues, with a positive correlation between the downregulation of let-7c and poor tissue differentiation in HCC [105]. Also, the expression of let-7g was downregulated in human HCC cell lines; in line with this finding, after transfection experiments in hepatocarcinoma cells, it was suggested that let-7g may act as tumor suppressor gene that inhibits HCC cell proliferation by downregulating c-Myc and upregulating the tumor suppressor gene p16(INK4A) [103], whereas other members of this family, such as let-7a/b, were found to be upregulated in human hepatocellular CSCs, representing possible molecular targets for eradication of hepatocarcinoma [97].

### 2.13. MiR-199a/b-3p

Recently, microarray and bioinformatics analyses, performed to reveal the specific miRNAs profile responsible for transition from steatosis to steatohepatitis in a rat model fed a fat-rich diet, showed the dysregulation of miR-199a during the progression of liver inflammation from steatosis, in which it is downregulated to steatohepatitis in which it is upregulated instead [102]. The miR-199a levels have been shown to be tightly related to the grade of liver fibrosis in both human and mouse, being significantly elevated in progressed liver fibrosis relative to healthy controls [72, 87]. Among the tumor suppressor miRNAs, miR-199a/b-3p represents the third most highly expressed in normal human liver and was found to be significantly downregulated in human HCC. Its low expression level was an independent predictor for reduced tumor-free survival in HCC patients, hence, confirming its importance in inhibiting HCC growth [32, 106].

### 2.14. MiR-128-2

MiR-128-2 is known as a proapoptotic miRNA, which negatively regulates cancer cell invasion and as an endogenous regulator of SIRT1 that in turn is able to affect also the p53 network [107, 108]. However, it has been recently identified a new role for miR-128-2 in the control of cholesterol homeostasis [109]. In this work, miR-128-2 was shown to regulate cellular cholestrol efflux by targeting the ATP-binding cassette transporters ABCA1 and ABCG1, as well as the retinoid X receptor *α* (RXR*α*) in hepatic cell lines and in tissues from mice fed a high-fat diet, in which its expression was downregulated. In this mechanism of action, miR-128-2 led to the synthesis and accumulation of intracellular cholesterol also by increasing the levels of SREBP-2, which in turn might stimulate its expression in a feed forward loop. Thus, it has been speculated that miR-128-2 reduction, observed in obese mice, might confer resistance to apoptosis and induce cancer. In conclusion, miR-128-2, by stimulating hypercholesterolemia that frequently occurs in obese patients, provides an important molecular link between obesity and cancer [109], which, however, warrants further investigation in a NAFLD scenario.

## 3. Conclusions and Future Perspectives

A better understanding of the molecular mechanisms underlying NAFLD pathogenesis and progression is a mandatory step to more effective, preventive, and curative treatments. To this aim, the identification of reliable and specific biomarkers is required. To date, only a few biomarkers have a potential diagnostic and prognostic relevance, and most of them can only be evaluated through invasive methods. The choice of optimal markers for HCC surveillance has to be determined by the etiology of the underlying chronic liver disease. Therefore, early diagnosis and monitoring of HCC, in the setting of NAFLD is of utmost importance. Correct assessment of the stage of NAFLD, by measuring the degree of liver inflammation and the staging of fibrosis, can also contribute to the efficacy of therapeutic interventions and to improved prognosis. Noninvasive methods ranging from circulating biomarker assays to advanced imaging techniques, such as Positron Emission Tomography-Computed Tomography (PET-CT) and ultrasound (e.g., Contrast-Enhanced Ultrasound (CEUS)), elastography (e.g., Fibroscan, and Acoustic Radiation Force Impulse (ARFI)), are being developed and ameliorated in this sense. Importantly, biomarkers detectable both in liver tissue and body fluids like serum, urine, and bile (e.g., preoperative serum VEGF), which are easily accessible and potentially useful also for an early diagnosis of tumor recurrence and metastasis, acquire a great relevance. Among them, miRNAs might occupy a paramount position, because their dysregulation showed high prognostic and predictive value in a broad spectrum of rodents and human pathological liver conditions, including NAFLD [31, 35–37, 55] and cancer [23, 29, 30]. Moreover, miRNAs expression patterns in human diseases appear to be tissue-specific [37, 110]; they possess unusually high stability in formalin-fixed tissues [111] and even in archived plasma samples [112]. For example, understanding their differential expression in the adipose tissue of obese patients may be useful to advance the comprehension of the molecular mechanisms important for the development of NAFLD/NASH, as well as to exploit them as diagnostic tools to distinguish patients with benign simple steatosis from those with steatohepatitis and thus to enable new targeted therapies. The possibility to assign a specific spectrum of miRNA expression to each stage of NAFLD will represent a fundamental breakthrough for identifying new important diagnostic and therapeutic tools to deal with the disease. In order to formulate panels of miRNAs as suitable molecular biomarkers, there is still the need for both standardized approach for their assessment and validation in larger cohorts of patients. The combination of various miRNAs, evaluable in peripheral tissues and body fluids, with more traditional histopathological characteristics, may further enhance their predictive ability in subjects at high risk of disease progression and recurrence. Besides, their use alongside the already adopted biomarkers may increase their prognostic value, with important consequences for patients and, in turn, for the public health system. On the one hand, many promising noninvasive tests are useful to distinguish cirrhosis from no or minimal fibrosis, though for the diagnosis of significant liver fibrosis noninvasive methods cannot yet replace liver biopsy. Furthermore, malignant transformation may occur regardless of the etiologic agent, through a pathway of increased liver cell turnover induced by chronic liver injury and regeneration, in a context of inflammation, immune response, and oxidative DNA damage [113]. Thus, the global miRNA expression profiling may represent the most appropriate technology for monitoring the progression of liver damage and exploring the heterogeneous origin of HCC. In fact, in a clinical setting, the recent microarray-based gene profiling technology allows the study of multiple genes and miRNAs simultaneously, providing important findings relating to the carcinogenic process. In addition, microarray analysis may enable to predict which patients are likely to respond to specific chemotherapeutic agents or combinations, thus creating individualized treatment regimes. Moreover, targeted therapy, which specifically inhibits molecular abnormalities, has recently emerged as a novel approach for the effective medical treatment of malignancies, including HCC [114, 115]. A combinatorial approach using different methods (such as* in silico* and biological analysis), rather than a single approach, may be recommended for providing a global overview also of the underlying disease mechanisms and derangements of molecular pathways occurring in NAFLD, so helping to describe its genetic architecture [116, 117]. It is crucial integrating experimental evidence with computational analysis, possibly collected using high-throughput technologies, to evaluate and identify both miRNAs expression and their specific targets, so as to have a better understanding of the biological impact of miRNA-mediated gene regulation under physiological and pathological conditions. In fact, the miRNA-mRNA interactions defined only by computational prediction may contain false-positive results in terms of “functional” pairs, because the two molecules may not be expressed together.

Hence, the integration between computational approaches (e.g., bioinformatics and biomodeling) and high-throughput genomic sequencing technologies for the identification of novel gene regulatory networks and prediction of miRNA targets, along with quantitative proteomic techniques, may represent the beginning of a new era in NAFLD as well as in cancer gene discovery, which promises to open up alternative avenues for their management. New combinative methods of systems biology, by helping to elucidate the underlying biological process and pathogenic pathways leading to NAFLD, may have a tremendous impact on the diagnosis and prevention of NAFLD in the near future. For instance, in a recent work [118] Sookoian and Pirola dissected distinctive disease-associated molecular signatures between NAFLD and alcoholic fatty liver disease (AFLD) in a comparative analysis. In their work they integrated genomic, molecular, and physiological data extracted by computational data mining using different platforms and software applications for gene enrichment analysis and protein-protein interaction networks to predict novel biomolecular interactions among genes and proteins associated with NAFLD/AFLD. Instead, Moylan and colleagues analysed and discriminated gene expression profiles in liver biopsies from low-risk, mild, and severe NAFLD patients (according to fibrosis stage) to identify processes that are differentially activated and that might represent novel biomarkers or therapeutic targets for the treatment of patients at high risk for NAFLD-related mortality [119]. These innovative methods for searching unknown loci and molecular targets involved in the pathogenesis of liver disease and their comprehensive analysis might also be exploited for the investigation of NAFLD-associated profiling of miRNAs and, through a systematic approach, for mining miRNA-target sites underlying disease progression. Furthermore, technological advances in the targeted delivery of miRNAs or synthetic miRNA mimics (e.g., through retroviral vectors, adeno-associated viral vectors, nanoparticles) [120, 121] and selective miRNA silencing strategies [122] will provide new hope and possibilities for their use as novel therapeutic agents against HCC, holding the potential for the treatment of NAFLD too. The emergence of circulating miRNAs as noninvasive diagnostic and prognostic markers offers a novel choice for using their expression profile in clinical practice. Nevertheless, their use as potential drug targets and therapeutics still faces numerous challenges, including specific target identification, mode of delivery, specificity, and length of action [123]. In addition, if circulating miRNAs have a cellular rather than an organ origin, as well as their* in vivo* biodistribution and their turnover are still open questions. Although many studies have shown molecular mechanisms underpinning the pathogenesis of NAFLD and related HCC development, they have not clarified all the interactions among different mediators and how they can influence systemic homeostasis. In fact, it has been shown that NAFLD is strongly associated with cancer-related pathways not only limited to the liver [118]; among other things, dysregulated expression of several miRNAs can also be due to disorders in different tissues/organs which may or may not be related to liver disease, entailing sometimes difficult interpretations by the clinicians. For example, miR199a, which was tightly related to steatohepatitis and liver fibrosis, when released into circulating blood was also found to be involved in acute myocardial infarction and upregulated during ischemia/reperfusion (I/R) injury [124]. Also, the complex feedback network composed by epigenetic pathways and miRNAs (including, among others, Let-7a) forms an important regulatory circuit, whose disruption underlies a variety of pathological phenotypes and cancers not always related to the liver, which may complicate our understanding of diseases [125]. These aspects may represent a significant limitation to the use of miRNAs as reliable biomarkers in the setting of metabolic disorders involving several organs and during the transition from NAFLD to HCC, which deserve additional exploration in human investigations. Lastly, even though circulating levels of miR-122, which plays multiple roles in liver homeostasis, are well accepted as robust markers for predicting NAFLD progression [31, 126], similarly to other circulating miRNAs, miR-122 is taken up also by other cells in distant tissues other than the one of origin, and it was suggested to be an interesting biomarker for cardiovascular disease (CVD), such as hypertension, in NAFLD patients. Mir-122 is known to regulate the expression of dozens of interacting genes simultaneously in different tissues, acting as a bridge to link metabolic and cardiovascular diseases [126]. Collectively, the balance between benefit/drawbacks about the role played by miRNAs concomitantly in different organs and their helpful potential for the screening of hepatic disease has to be thoroughly considered and investigated with the aim to improve patients' diagnosis. Therefore, further studies addressing these issues will be essential prior to definitive clinical trials but, all things considered, we think that miRNAs could represent a powerful tool in the hands of clinicians to improve the diagnosis and prognosis of NAFLD.

## Figures and Tables

**Table 1:** Descriptive summary of dysregulated miRNAs in NAFLD.

Disease	Modulation		
	Upregulated	Downregulated	References
Steatosis/NASH	Pri-miR-26a-1 (in NASH), miR-122, miR-370, miR-34a, miR-16, miR-33a/b, miR-200a/b, miR-429, miR-221, miR-155, miR-181a, miR-10b, let-7b (in NASH), miR-199a (in NASH)	miR-99a/b, miR-21, miR-29c, miR-451, let-7d (in NASH), miR-199a (in steatosis), miR-128-2	[31, 35, 36, 38–40, 43, 45, 46, 52–55, 60, 65, 67, 68, 71, 76, 77, 98, 102, 109]

Fibrosis	miR-122, miR-34a, miR-199a/a*, miR-200a/b, miR-21, miR-221/222, miR-155, let-7e	miR-16, miR-15b, miR-99b, miR-197, miR-29	[31, 39, 45, 55, 66, 72, 82, 86, 87]

Cirrhosis	miR-34a, miR-21, miR-31, miR-122, miR-221, miR-155, miR-181b	miR-29	[30, 31, 85, 86, 91–93]

HCC	miR-122 (serum), miR-16, miR-33, miR-21, miR-31, miR-221/222, miR-155, miR-181a/b, let-7a/b, miR-10b	miR-122 (tissue), miR-34a, miR-200a/b, miR-99a, let-7c/g, miR-199a/b-3p	[29, 33, 40, 47, 48, 50, 51, 56, 57, 62, 65, 70, 73–75, 78, 81, 83–85, 88–92, 94–97, 99, 103, 105, 106]

NASH: nonalcoholic steatohepatits; HCC: hepatocellular carcinoma.
